# Supplementary material for: Antigen Unmasking Is Required to Clinically Assess Levels and Localisation Patterns of Phospholipase C Zeta in Human Sperm
Source: Pharmaceuticals (Basel). 2023 Jan 28;16(2):198. doi: 10.3390/ph16020198 (PMC9962097; doi:10.3390/ph16020198)
Supplement: Supplementary file 1 [file pharmaceuticals-16-00198-s001.zip › pharmaceuticals-2118778-supplementary.pdf]

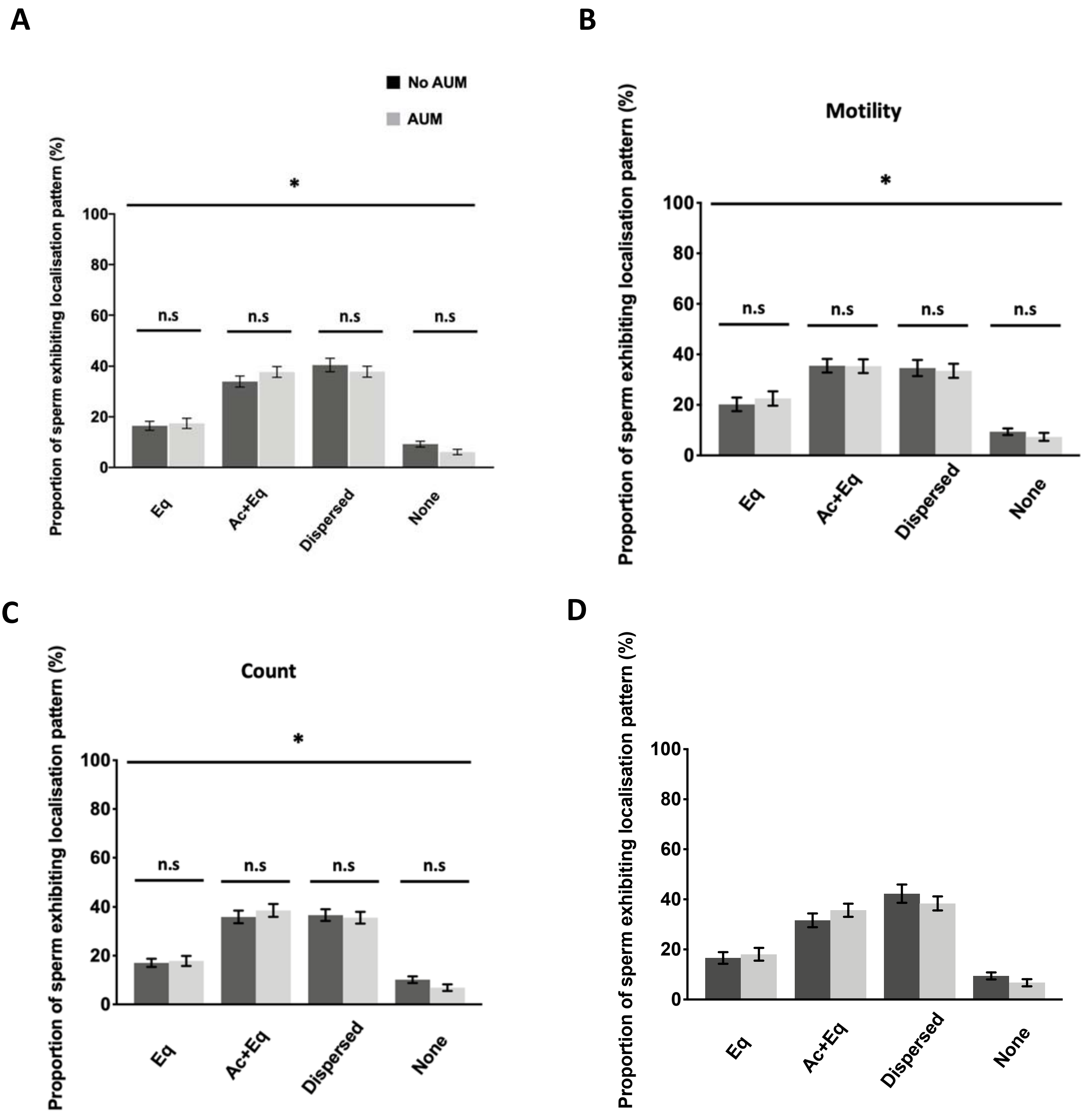

Supplementary Figure S1: Representative histograms indicating the change in proportions (%) of sperm exhibiting PLCζ localisation patterns with (light bars) and without (dark bars) antigen unmasking (AUM and no AUM, respectively) in A) sperm from all patients collectively, and in optimal parameters of B) sperm motility (≥40%), C) sperm concentration (count) (≥$15x10^6$ sperm/ml), and D) semen volume (1.5-5.5ml). AUM did not exert any significant change between any pattern of localisation observed throughout all optimal sperm parameters examined. Data are indicative of 100 cells examined from 55 patients each for both AUM and no AUM groups. Asterisks (*) indicate a statistically significant (*p*≤0.05) difference, while n.s indicates a statistically non-significant difference (*p*>0.05).

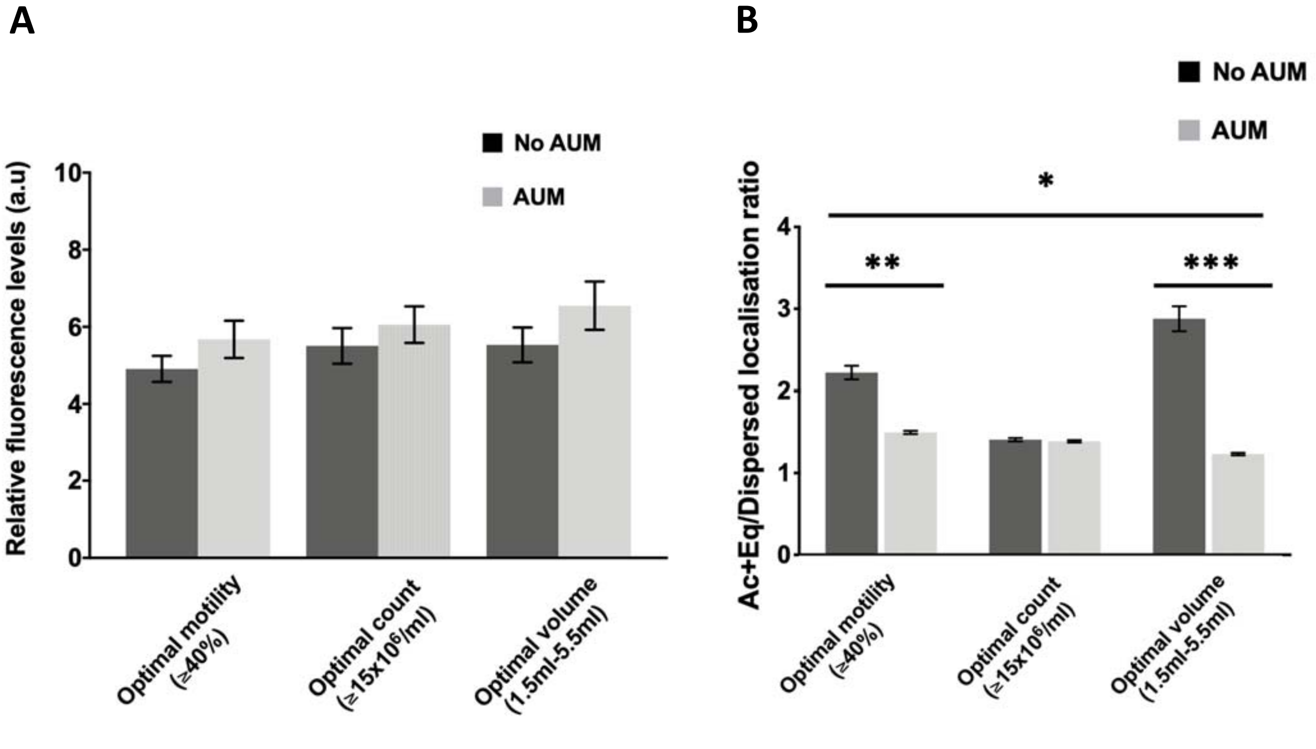

Supplementary Figure S2: Representative histograms indicating the change in A) relative fluorescence levels and B) acrosomal+equatorial/dispersed localisation pattern ratio of PLCζ without (dark bars) and with (dark bars) antigen unmasking (no AUM and AUM, respectively in optimal parameters of sperm motility (≥40%), sperm concentration (count) (≥15x10^{6} sperm/ml), and semen volume (1.5-5.5ml). Asterisks (*,**,***) indicate a statistically significant (p≤0.05) difference. Data are indicative of 100 cells examined from 55 patients each for both AUM and no AUM groups. a.u: arbitrary units.

Supplementary Table S1: Correlative analysis between the change in relative fluorescence levels and acrosomal+equatorial (Ac+Eq) and dispersed localization (Ac+Eq/Dispersed) ratio with parameters of human patients undergoing fertility treatment, indicating the lack of statistical significance between parameters examines ($p > 0.05$). a.u: arbitrary units.

	Motility (%)	Count (10^6/ml)	Volume (ml)	Male age (years)	Female age (years)
**Change in relative fluorescence following AUM (a.u)**	*p=0.3029*	*p=0.1066*	*p=0.4148*	*p=0.1991*	*p=0.3091*
**Change in Ac+Eq/Dispersed ratio following AUM (a.u)**	*p=0.0718*	*p=0.2619*	*p=0.1942*	*p=0.3069*	*p=0.3847*

Supplementary Table S2: Mean (±standard deviation) values of motility (%), Count (106/ml), and volume (ml) of sperm from males included in the study (n=55).

	**Motility (%)**	**Count (10^6/ml)**	**Volume (ml)**
**Mean (± SD)**	44 (±22.4)	77.4 (±65.7)	3.1 (±1.5)
